# Is Macronutrients Intake a Challenge for Cardiometabolic Risk in Obese Adolescents?

**DOI:** 10.3390/nu12061785

**Published:** 2020-06-16

**Authors:** Sara Vizzuso, Matilde Amatruda, Alberico Del Torto, Enza D’Auria, Giulio Ippolito, Gian Vincenzo Zuccotti, Elvira Verduci

**Affiliations:** 1 Department of Health Sciences, University of Milan, 20133 Milan, Italy; sara.vizzuso@unimi.it (S.V.); matilde.amatruda@unimi.it (M.A.); giulio.ippolito@unimi.it (G.I.); 2 Centro Cardiologico Monzino IRCCS, 20138 Milan, Italy; alberico.deltorto@cardiologicomonzino.it; 3 Department of Pediatrics, Vittore Buzzi Children’s Hospital University of Milan, 20154 Milan, Italy; enza.dauria@unimi.it (E.D.); gianvincenzo.zuccotti@unimi.it (G.V.Z.)

**Keywords:** adolescent obesity, fats intake, macronutrients, metabolic syndrome, cardiometabolic risk

## Abstract

(1) Background: Pediatric obesity is an emerging public health issue, mainly related to western diet. A cross-sectional study was conducted to explore the association between macronutrients intake and cardiometabolic risk factors in obese adolescents. (2) Methods: Ninety-three Italian obese adolescents were recruited; anthropometric parameters, body composition, glucose and lipid metabolism profiles were measured. Macronutrients intake was estimated by a software-assisted analysis of a 120-item frequency questionnaire. The association between macronutrients and cardiometabolic risk factors was assessed by bivariate correlation, and multiple regression analysis was used to adjust for confounders such as age and sex. (3) Results: By multiple regression analysis, we found that higher energy and lower carbohydrate intakes predicted higher body mass index (BMI) z-score, *p* = 0.005, and higher saturated fats intake and higher age predicted higher HOmeostasis Model Assessment of insulin resistance (HOMA-IR) and lower QUantitative Insulin-sensitivity ChecK (QUICK) index, *p* = 0.001. In addition, a saturated fats intake <7% was associated with normal HOMA-IR, and a higher total fats intake predicted a higher HOMA of percent β-cell function (HOMA-β), *p* = 0.011. (4) Conclusions: Higher energy intake and lower carbohydrate dietary intake predicted higher BMI z-score after adjustment for age and sex. Higher total and saturated fats dietary intakes predicted insulin resistance, even after adjustment for confounding factors. A dietary pattern including appropriate high-quality carbohydrate and reduced saturated fat intakes could result in reduced cardiometabolic risk in obese adolescents.

## 1. Introduction

Obesity is a chronic condition characterized by an abnormal or excessive fat accumulation that presents a risk to health. From 1980 to nowadays, adult and childhood obesity has become a priority health issue in terms of prevalence and economic burden [1,2,3]. During the last three decades, in both developed and developing countries, obesity prevalence rates increased about 27% in adults and 47% in children, for a total of 2.1 billion individuals considered overweight or obese [4]. Such an alarming spread is mainly due to unhealthy westernized dietary habits within an “obesogenic environment” that promotes a sedentary lifestyle [5]. According to WHO data (2016), 41 million children under 5 years and over 340 million children and adolescents are overweight or obese. The WHO European Childhood Obesity Surveillance Initiative (COSI, fourth edition) reported an obesity prevalence among school-aged European children (6–9 years) between 4% and 21% for boys and between 2% and 19% for girls [6]. Obesity induces a chronic, low-grade inflammation that leads to vascular dysfunction, thrombotic disorders, and metabolic alterations resulting in hypertension, dyslipidemia, and insulin resistance [7]. Although it was thought to be an adult-onset disease, metabolic syndrome (MetS) is becoming increasingly prevalent also in childhood and adolescence [8]. In 2007, the International Diabetes Federation (IDF) suggested a consensus definition of MetS in children and adolescents: for adolescents aged 10 years or older, metabolic syndrome is diagnosed with abdominal obesity (waist circumference >90° percentile) in the presence of two or more other clinical features between elevated triglycerides (TG ≥ 150 mg/dL), low high-density lipoprotein cholesterol (HDL-c ≤ 40 mg/dL), high blood pressure (systolic (SBP) ≥ 130 mmHg, diastolic (D)BP ≥ 85 mmHg), increased plasma glucose (fasting glucose ≥ 100 mg/dL). For adolescents older than 16 years, adult criteria can be used [9]. Several epidemiological studies showed that MetS is not just a simple cluster of metabolic complications related to excess adipose tissue but represents an important cardiovascular risk factor itself [10].

Given the limited indications for pharmacologic treatment, encouraging healthy eating and lifestyle is a crucial key in preventing and managing pediatric obesity [11,12]. A recent Cochrane systematic review proved with moderate quality evidence that a multidisciplinary intervention including diet, physical activity, and lifestyle education can be useful in overweight or obese adolescents, mainly when compared with no treatment or waiting list controls [13]. Since pediatric obesity is an emerging and serious public health concern, the aim of this study was to explore the association between macronutrients intake and cardiometabolic risk factors in a cohort of Caucasian obese adolescents and to suggest a dietary intervention targeted at lowering the cardiometabolic risk of obese adolescents.

## 2. Materials and Methods

### 2.1. Study Population

A descriptive, cross-sectional study was conducted in a cohort of 93 (37 males and 56 females) Italian obese patients, aged 10–17 years, recruited from January 2014 to January 2019 by our Department. Obesity was defined according to WHO criteria for children and adolescents aged 5–19 years [14]. All patients known to have any metabolic disease or taking drugs that could potentially affect the study outcomes were previously excluded. Parents of eligible patients or their legal guardian received detailed explanation about the aim of the study and signed a consent form. The Hospital Ethics Committee approved the study protocol and gave ethical clearance.

### 2.2. Measurements

#### 2.2.1. Anthropometrics

Body weight and height were measured using a mechanical column scale (seca 711; seca GmbH & KG, Hamburg, Germany) with an integrated measuring rod (seca 220; seca GmbH & KG, Hamburg, Germany). Body mass index (BMI) was calculated as weight/height^2^ (kg/m^2^). BMI z-score was assessed according to WHO curves specific for age and sex. Waist circumference (WC) was measured trough seca measuring tape 203 (seca GmbH & KG) at midpoint between costal margin and iliac crest, in a standing position, at the end of a gentle expiration [15,16]. Tricipital skinfold thickness was measured on the left side of the body, using a Harpenden Skinfold plicometer (Chasmors Ltd., London, United Kingdom) at the midpoint between acromion process and olecranon process [17].

Waist-to-height ratio (WHtR), an index of central adiposity, was calculated by dividing the WC by the height [18]. A WHtR value over 0.60 has been recently linked to a higher risk for MetS in adolescents [19].

Fat mass (FM), FM percentage (FM%), fat-free mass (FFM) and fat-free mass percentage (FFM%) were estimated using a bioelectrical impedance analysis system (BC 418 MA, Tanita Corp, Tokyo, Japan) [20]. An oscillometric device was used to check blood pressure (BP), according to the National High Blood Pressure Education Program Working Group recommendations [21].

#### 2.2.2. Biochemical Assessments

Blood samples were collected in standardized conditions: from 8:30 to 9, after 12 h of fasting, by cubital vein puncture. They were immediately sent to our clinical laboratory and analyzed to check for total cholesterol (TC), HDL-c, low-density lipoprotein cholesterol (LDL-c), TG, insulin, fasting glucose, glycated hemoglobin (HbA1c). US National Heart, Lung, and Blood Institute (NHLBI) lipid cutoff values, based on US normative data, were used to detect dyslipidemia [22]. Insulin, fasting glucose, and HbA1c levels were reported to our Clinical Laboratory range values.

#### 2.2.3. Dietary Habits

Subjects’ dietary habits were assessed through a food frequency questionnaire (FFQ) developed in 1990 at our Department, based on the original Block-FFQ [23] and revised in 2008 according to the full-length Block 2005 FFQ © (NutritionQuest, Berkeley, CA, USA). The FFQ is the most common method for dietary assessment used in large epidemiological studies [24]. The questionnaire consists of a list of 120 foods and beverages with response categories to indicate usual (daily, weekly, or monthly) frequency of consumption and portion (full, half, or double portion). Parents filled out the questionnaire about the patients’ dietary habits during a 50 min interview held by a trained dietitian. Usual portion sizes were estimated using household measures and the weight (e.g., pasta) or unit (e.g., fruit juice) of the purchase. A 24 h recall was additionally recorded at the end of the interview to standardize the usual serving size.

Energy intake analysis and nutrient quantification were performed using an ad hoc PC software program (MetadietaVR, 2013; METEDAsrl, via S.Pellico 4, San Benedetto del Tronto, AP, Italy). The food consumption frequency for each item was converted to daily intake and compared to age- and sex-specific Italian national dietary reference values for energy and nutrients [25].

#### 2.2.4. Cardiometabolic Risk Assessment

HOmeostasis Model Assessment of insulin resistance (IR) index (HOMA-IR), HOMA of percent β-cell function (HOMA-β%), and QUantitative Insulin-sensitivity ChecK Index (QUICK index) calculated on fasting samples are useful tools in the clinical practice to identify subjects at risk for type 2 diabetes mellitus [26].

Although the glucose clamp method remains the reference standard for a direct measurement of insulin sensitivity, these quick and simple indexes are ideal for large and longitudinal studies and more acceptable to children and adolescents [27].

HOMA-IR: HOMA-IR is the most widely used measure of insulin resistance; it is calculated as the product of fasting plasma insulin (µU/mL) by fasting plasma glucose (mmol/L) divided by 22.5 [28].

HOMA-IR varies according to age and gender. Recently, HOMA-IR reference ranges have been published for a large population of normal-weight and obese young Caucasians. According to Shashaj et al., an HOMA-IR value above the 75th percentile in obese participants identifies adolescents with cardiometabolic risk factors [26].

HOMA-β: HOMA-β is an index of β-cell function, calculated from fasting glucose and insulin or C-peptide concentrations as follows:

HOMA-β = (20 × fasting insulin in µU/mL)/(fasting glucose in mmol/L − 3.5) [29].

QUICK index: QUICK index is considered as a surrogate measure of insulin sensitivity [30].

It is calculated as 1/(log10 fasting plasma insulin in µU/mL + log10 glucose in mg/dL), considering as a reference the value of 0.37 ± 0.04 [30,31].

Triglyceride–Glucose index (TyG index): TyG index mainly reflects muscles’ resistance to insulin action [32] and it is calculated as ln [fasting triglycerides (mg/dL) × fasting glucose (mg/dL)/2].

Triglycerides/HDL ratio (TG/HDL): Children and adolescents at risk of atherogenic dyslipidemia and Impaired Fasting Glucose (IFG) have a value of triglycerides (mg/dL)/HDL cholesterol (mg/dL) ≥ 2.2 [33,34].

Atherogenic index of plasma (AIP): AIP is calculated as Log [TG (mg/dL)/HDL cholesterol (mg/dL)]; it reflects the relationship between protective and atherogenic lipoproteins and may predict the risk of cardiovascular diseases in adults and adolescents [35].

Visceral adiposity index (VAI): VAI reflects fat distribution and metabolism and is calculated as:

$$VAI\;\left(males\right)=\frac{WC}{39.68+\left(1.88\times BMI\right)}\times \frac{TG}{1.03}\times \frac{1.31}{HDL}$$

$$VAI\;\left(females\right)=\frac{WC}{39.58+\left(1.89\times BMI\right)}\times \frac{TG}{0.81}\times \frac{1.52}{HDL}$$

It is a useful tool for detecting MetS in children and adolescents [36].

### 2.3. Statistical Analysis

Descriptive data are reported as mean and standard deviation (SD) or median and min–max range, as appropriate. Normality tests were conducted to assess whether the variables were normally distributed. The association between macronutrients and cardiometabolic risk factors was assessed by Pearson’s or Spearman’s bivariate correlation coefficient, as appropriate. Forward stepwise multiple regression analysis was performed to predict each cardiometabolic risk factor from models including energy intake, amount of each macronutrient, age, and sex. Logarithmic transformation was performed on skewed data. Pearson’s chi-squared test was used to assess the association between normal saturated fats intake and HOMA-IR. All values of *p* ≤ 0.050 were considered to indicate statistical significance (two-tailed test). The statistical package for social sciences (SPSS) package version 20.0 (SPSS Inc., Chicago, IL, USA) was used for statistical analysis.

## 3. Results

### 3.1. Anthropometric Parameters in Obese Adolescents

Mean age at recruitment was 11 years. Fifty-six patients were females (60%). All patients were in pubertal stage. The median value of BMI z-score was 2.5. All patients had a waist circumference (median 91 cm) over 90° percentile for age and sex [16]. The median value of WHtR, an index of central adiposity, resulted higher than normal [37]. In 45 patients (48%), WHtR values over the cutoff of 0.6 were found. Eighty (92%) adolescents had a tricipital skinfold measure over 95° percentile for age and sex [38]. Data from bioimpedance segmental body composition analysis revealed that 85 patients (91.3%) had an FM% consistent with obesity, according to sex- and age-specific curves for body fat (Table 1) [39].

### 3.2. Macronutrients Intake in Obese Adolescents

In our cohort, the median energy intake, adjusted for sex and age, was normal compared to national dietary reference values (DRVs). The average daily protein intake was higher than the reference value (91 g/day vs. 39–50 g/day). The median lipid, saturated fat, and carbohydrate intakes (%) were within the reference range. Sugar intake (%) was higher than the reference value (18% vs. 15%). The intake of polyunsaturated fats was below the reference value (Table 2).

### 3.3. Glucose and Lipid Metabolism in Obese Adolescents

The median values of fasting glucose, insulin, and HbA1c serum levels were within the normal values. IFG was found in 3.2% of the patients, and Impaired Glucose Tolerance (IGT) in 2.1% of them. Thirteen patients (about 14%) had HbA1c levels consistent with prediabetes. No one was diagnosed with diabetes type 1 in our population. In 60.2% of the patients, the HOMA-IR value was above the 75th percentile of the reference population and therefore associated with an increase of cardiometabolic risk (Table 3) [26].

The median values of TC, LDL-c, HDL-c, TG resulted normal compared to the reference values (Table 4). The TG/HDL ratio and AIP (SD) were calculated (Table 4). TC borderline–high values were found in 18 (19%) patients, and high values in 5 patients (5.3%). LDL borderline high values were found in 10 patients (11%), high LDL values were found in 5 patients (5%); 21 patients (22%) had HDL-c borderline low values, 27 patients (29) had low HDL values. Sixteen patients (17%) had TG borderline high values, 25 patients (27%) had high TG values.In our cohort, 13 patients (14%) were diagnosed with MetS, according to IDF criteria.

### 3.4. Association between Macronutrients and Cardiometabolic Risk Factors

By bivariate correlation, energy intake and total fats intake were both significantly and positively associated with BMI z-score and HOMA-β. Protein intake was positively associated with BMI z-score and HOMA-IR and negatively associated with the QUICK index. Saturated fats intake was positively associated with HOMA-IR and HOMA-β and negatively associated with the QUICK index. Carbohydrates percentage was inversely related to BMI z-score and HOMA-β. No significant association was found between macronutrients intake and TyG index, TG/HDL, AIP, and VAI (Table 5).

Multiple regression was performed to predict the BMI z-score from energy intake and macronutrients intake after adjusting for age and sex. A model comprising energy intake and carbohydrate intake predicted the BMI z-score. Energy intake was positively associated with BMI z-score, while carbohydrate intake (g) was negatively associated with it (Table 6).

A model comprising saturated fats intake (g) and age predicted HOMA-IR, *p* = 0.001, adj. R^2^ = 0.123. Both variables added statistically significantly to the prediction, *p* < 0.050 (Table 7). Patients with normal saturated fats intake according to age- and sex-specific Italian national dietary reference values for energy and nutrients (<10% of total energy, E, [25]) did not have a significantly higher probability of having a normal HOMA-IR. On the contrary, patients with saturated fats intake <7% of E (as recommended by the National Lipid Association Expert Panel on Familial Hypercholesterolemia [40]) had a significantly higher probability of having a normal HOMA-IR, *p* = 0.050 (Table 8).

Only total fats intake (g) predicted HOMA-β after adjusting for energy intake, other macronutrients intake, age, and sex; *p* = 0.011, adj. R^2^ = 0.060 (Table 9).

A model comprising age and saturated fats intake predicted the QUICK index using multiple regression analysis, F (2, 90) = 7.271, *p* = 0.001, adj. R^2^ = 0.120. Both variables added statistically significantly to the prediction, *p* < 0.050 (Table 10).

By multiple regression analysis, a model containing energy intake, macronutrients intake (g), age, and sex could not predict TyG index, TG/HDL ratio, AIP, or VAI.

## 4. Discussion

Thirteen adolescents (14%) were diagnosed with MetS in our population. This finding is comparable to the overall prevalence of MetS in other cross-sectional studies conducted in obese adolescents, with rates ranging from 10% to 38% [41,42,43]. The real prevalence of this condition in children and adolescents is hard to estimate due to the lack of a consensus on its definition [44].

In our study, energy intake and carbohydrate dietary amount predicted BMI z-score after adjustment for age and sex, while protein and total fats intakes were predictors only in unadjusted analysis. Therefore, our data suggest that diets with higher energy intake and lower carbohydrate content are a substantial risk factor for developing obesity in adolescents. Adolescent obesity management requires complex efforts, both with lifestyle and behavioral interventions and with nutritional support. The results of our study suggest that a nutritional intervention in obese adolescents should be focused on reducing the energy intake, in agreement with current guidelines that suggest consuming fewer calories, fewer sugars, less saturated fats, more unsaturated fats (including vegetable oils) and eating vegetables and fruits daily [45]. According to a recent metanalysis of nutritional intervention studies among obese children and adolescents, weight reduction can be achieved with a low-energy intake diet, independently of macronutrients distribution [46]. However, our data also suggest that a higher carbohydrate intake is protective against adolescent obesity. Data on optimal carbohydrates intake in adolescents are lacking. A recent systematic review and meta-analysis of eleven observational studies enrolling a total of 153891 obese adults investigated the relationship between carbohydrate intake and obesity. Six studies linked a higher carbohydrate intake to a reduced risk of obesity, while five studies showed an increased risk, with the pooled odds ratio being non-significantly different from 1. The authors suggested that these inconclusive results could be due to differences in the quality of the carbohydrates (refined vs. unrefined) mainly represented in the different studies, and this view seems supported by the literature showing an increased risk of obesity conferred by diets rich in refined sugars, as opposed to diets rich in unrefined sugars [47]. Moreover, diets rich in refined, high-glycemic-index carbohydrates have been linked to an increased risk of myocardial infarction in adults [48].

Moreover, in our cohort of obese adolescents, higher total and saturated fats dietary intakes predicted insulin resistance, even after adjustment for age and sex. An increased intake of total fats was associated with a raised HOMA-β, while an elevated intake of saturated fats was associated with a raised HOMA-IR and a reduced QUICK index. A causal link between total and saturated fats dietary intakes and altered glucose metabolism could be traced in the pro-inflammatory effect of dietary fats. In a cohort of 219 girls aged 12 to 17 years, a western dietary pattern as opposed to a Mediterranean dietary pattern was associated with reduced levels of adiponectin [49]. A cross-sectional study including 532 European adolescents also found that high sugar and fat dietary intakes were associated to higher levels of inflammation [50]. Studies addressing the association between macronutrients intake and glucose metabolism among obese adolescents are scarce. In a large cohort of adolescents (with subjects included independently of their BMI), an energy-dense, high-fat, and low-fiber dietary pattern was positively associated with higher levels of insulin and HOMA-IR [51]. Moreover, a recent Brazilian cross-sectional study found that in a cohort of adolescents (again, including subjects independently of their BMI), an elevated saturated fat intake was associated with insulin resistance [52]. In adults, different studies on large cohorts and with long follow-up have demonstrated that a low-fat (but high-carbohydrate) diet is effective in the prevention of type 2 diabetes mellitus, reporting reductions in its incidence of up to 58% [53,54,55,56]. In particular, a recent systematic review and meta-analysis of observational studies in adults showed that the effect on the cardiovascular risk of replacing saturated fats in the diet depends on the type of quality carbohydrate used for replacement. Only the replacement with low-glycemic-index carbohydrates is able to decrease the risk [57]).

According to our knowledge, few studies evaluated the association between macronutrients intake and cardiometabolic risk factors in obese adolescents, and in this view our results could represent a novelty in the field.

## 5. Conclusions

Our results are hypothesis-generating and suggest that in obese adolescents, a dietary intervention targeted at reducing calories intake while increasing high-quality carbohydrates intake (but with limited sugars) could reduce the ponderal excess. Moreover, lowering fat intake could result in improved glucose tolerance, reduced risk of developing diabetes mellitus, and thus, reduced cardiometabolic risk in adulthood. In particular, dietary patterns with saturated fats intake <7% E (according to the heart-healthy fat-modified diet [40]) could be considered for obese adolescents to reduce their cardiovascular risk. Further studies are therefore needed to assess the impact of nutritional interventions on cardiometabolic risk factors in adolescents.

### Limitations

Our study has some limitations, including its observational nature and the lack of a control group for ethical reasons, considering blood sampling as an invasive procedure for healthy adolescents. Besides, the number of study participants was limited; therefore, the results of our analyses need to be replicated in a larger cohort. The cross-sectional design could not determine causal relationships between the analyzed variables. Physical activity was not assessed, and therefore the analyses did not include it. In addition, there are no unique definitions for metabolic syndrome and cardiometabolic risk assessment in adolescents. Another limitation is that the gold standard to assess food intake is the 3-day food record; however, the Frequency Food Questionnaire is largely used and in the present study was associated with a 24 h recall to standardize the usual serving size. Moreover, data regarding the socioeconomic and educational levels of the families, their knowledge of healthy diet, and their access to quality food are lacking.

## Figures and Tables

**Table 1:** Anthropometric parameters. BMI, body mass index, WHtR, waist-to-height ratio, FM, fat mass, FFM, fat-free mass.

Variable	Mean (SD)	Median (25–75° pc)
Age (y)	11 (1)	12 (10–12)
BMI z-score	2.7 (0.6)	2.5 (2.25–2.96)
Waist circumference (cm)	91.7 (9)	91 (86–97)
Tricipital skinfold (mm)	31.6 (6)	31.4 (27.4–36.7)
WHtR	0.6 (0.1)	0.59 (0.56–0.63)
FM (g)	27,420 (9097)	25,300 (20,200–32,600)
FFM (g)	42,424 (8764)	41,100 (36,800–47,100)
FM (%)	38 (6)	37 (34–42)
FFM (%)	61 (6)	62 (58–65)
Systolic blood pressure (mmHg)	116 (11)	114 (110–123)
Diastolic blood pressure (mmHg)	62 (8)	61 (57–66)

**Table 2:** Daily dietary intake of energy and macronutrients.

Variable	Mean (SD)	Median (25–75° Centile)	Reference Values [24]
Energy (kcal/day)	2358 (836)	2243 (1692–2868)	Boys: 2340–3480 kcal/day (AR)
			Girls: 2120–2690 kcal/day (AR)
Proteins (g/day)	102 (44)	91 (71–122)	Boys: 39–50 g/day (AR)
			Girls: 39–40 g/day (AR)
%	17 (5)	17 (15–20)	12–18% En (RI)
Total fats (g/day)	88 (44)	83 (58–109)	
%	30 (8)	31 (27–35)	20–35% En (RI)
Saturated fats (g/day)	22 (10)	19 (15–25)	<10% En (SDT)
%	8.2 (3)	8 (7–25)	
Monounsaturated fats (g/day)	33 (14)	31 (22–41)	
%	12 (5)	12 (9–15)	
Polyunsaturated fats (g/day)	10 (5)	9 (7–12)	
%	4 (2)	4 (3–4)	5–10% En (RI)
Carbohydrates (g/day)	309 (115)	294 (223–375)	
%	48 (11)	50 (46–54)	45–60% En (RI)
Sugars (g/day)	106 (50)	94 (73–134)	
%	18 (6)	17 (14–22)	<15% En (SDT)
Soluble Fiber (g/day)	5 (2)	5 (3–6)	8.4 g/1000 kcal (AI)
Insoluble Fiber (g/day)	9 (5)	9 (6–13)	

Values are expressed as mean (SD) and median (25th–75th percentile).

**Table 3:** Glucose metabolism in obese adolescents. HbA1c, glycated hemoglobin, HOMA-IR, HOmeostasis Model Assessment of insulin resistance IR, QUICK, QUantitative Insulin-sensitivity ChecK, HOMA-β, HOMA of β-cell function, TyG, Triglyceride–Glucose.

Variable	Mean (SD)	Median (25–75° Centile)	Normal Values
Glucose (mg/dL)	84 (7.5)	84 (79–89)	<100
HbA1c (mmol/mol)	34.9 (3.5)	35 (33–37)	20–42
HOMA-IR	4.2 (2.9)	3.7 (2.4–5.4)	<75° pc for sex and age in obese young Caucasian according to [26]
HOMA-β	374 (266)	305 (230–420)	
QUICK index	0.32 (0.02)	0.32 (0.30–0.33)	
TyG index	4.5 (0.29)	4.5 (4.3–4.6)	8 [32]

Values are expressed as mean (SD) and median (25th–75th percentile).

**Table 4:** Lipidic profile in obese adolescents. LDL, low-density lipoprotein, HDL, high-density lipoprotein, TG, triglycerides, AIP, atherogenic index of plasma.

Variable	Mean (SD)	Median (25–75° Centile)	Reference Values
Total Cholesterol (mg/dL)	153 (26)	151 (132–169)	≤200 mg/dL [22]
LDL Cholesterol (mg/dL)	91 (23)	89 (76–105)	≤130 mg/dL [22]
HDL Cholesterol (mg/dL)	46 (11)	45 (39–54)	>40 mg/dL [22]
Triglycerides (mg/dL)	121 (102)	88 (67–133)	<130 mg/dL [22]
TG/HDL	3.0 (3.5)	1.9 (1.3–3.2)	≤2.2 [33,34]
AIP	0.4 (0.9)	0.3 (0.1–0.5)	

Values are expressed as mean (SD) and median (25th–75th percentile).

**Table 5:** Association between macronutrients and cardiometabolic risk factors (ratio coefficient and statistical significance. VAI, visceral adiposity index. *p*-values ≤ 0.050 are shown in bold.

	BMI Z-Score	HOMA-IR	HOMA-β	QUICK Index	TyG Index	TG/HDL	AIP	VAI Index
Energy (kcal)	0.217 0.037 *	0.152 0.146	0.237 0.022 *	−0.170 0.104	0.117 0.262	0.023 0.825	0.027 0.796	0.037 0.728
Proteins (%)	−0.009 0.929	0.169 0.106	0.111 0.290	−0.162 0.121	0.032 0.762	0.088 0.399	0.088 0.403	0.068 0.520
Proteins (g)	0.240 0.020 *	0.224 0.031 *	0.249 0.016 *	−0.235 0.023 *	0.134 0.201	0.038 0.716	0.042 0.691	0.038 0.714
Total fats (%)	0.130 0.215	0.110 0.295	0.203 0.051	−0.110 0.293	−0.023 0.823	−0.029 0.783	−0.029 0.780	0.076 0.467
Total fats (g)	0.291 0.005 *	0.129 0.218	0.288 0.005 *	−0.143 0.172	0.157 0.132	0.080 0.445	0.083 0.427	0.150 0.152
Saturated (%)	−0.112 0.284	0.227 0.028 *	0.148 0.158	−0.210 0.043 *	0.046 0.660	0.051 0.625	0.051 0.626	0.044 0.678
Saturated (g)	0.133 0.204	0.268 0.009 *	0.278 0.007*	−0.274 0.008 *	0.137 0.192	0.039 0.711	0.042 0.690	0.074 0.482
Monounsaturated (%)	−0.007 0.950	0.241 0.020 *	0.200 0.055	−0.213 0.040 *	−0.009 0.931	0.015 0.889	0.013 0.900	0.048 0.645
Polyunsaturated (%)	−0.003 0.978	0.031 0.769	0.106 0.313	−0.003 0.976	0.023 0.830	0.106 0.313	0.108 0.305	0.101 0.334
Carbohydrates (%)	−0.299 0.004 *	−0.182 0.081	−0.276 0.007 *	0.177 0.089	−0.101 0.336	−0.061 0.559	−0.061 0.564	−0.157 0.134
Carbohydrates (g)	0.050 0.639	0.046 0.662	0.122 0.245	−0.066 0.531	0.046 0.663	−0.037 0.724	−0.034 0.747	−0.042 0.694
Sugars (g)	0.137 0.191	0.015 0.888	0.160 0.126	−0.031 0.766	0.082 0.433	0.040 0.703	0.044 0.673	0.027 0.797
Sugars (%)	−0.072 0.493	−0.199 0.056	−0.057 0.589	0.186 0.074	−0.019 0.860	0.025 0.812	0.026 0.807	−0.003 0.975
Insoluble fiber (g)	0.016 0.880	0.026 0.807	0.070 0.504	−0.035 0.737	0.081 0.438	0.024 0.821	0.022 0.833	0.011 0.917
Soluble fiber (g)	0.036 0.734	−0.038 0.717	0.057 0.589	0.027 0.798	0.045 0.669	−0.002 0.982	−0.003 0.975	0.002 0.983

* statistically significant association (*p* < 0.05).

**Table 6:** Multivariable model predicting the BMI z-score, F (2, 90) = 5.652, *p* = 0.005, adj. R^2^ = 0.092. *p*-values ≤ 0.050 are shown in bold.

	B	Std. Error	t	Sig.
(Constant)	−0.163	0.206	−0.794	0.430
Energy intake, kcal (log)	0.383	0.115	3.327	**0.001**
Carbohydrate, g (log)	−0.285	0.109	−2.621	**0.010**

**Table 7:** Multivariable model predicting HOMA-IR, F (2, 90) = 7.433, *p* = 0.001, adj. R^2^ = 0.123. *p*-values ≤ 0.050 are shown in bold.

	B	Std. Error	t	Sig.
(Constant)	–1.464	0.592	–2.474	**0.015**
Age, years (log)	1.525	0.539	2.830	**0.006**
Saturated fats, g (log)	0.295	0.121	2.427	**0.017**

**Table 8:** Association of normal HOMA-IR and saturated fats intake <10% of total energy (E, χ^2^ 2.461, *p* = 0.117) or <7% E (χ^2^ 3.840, *p* = 0.050).

	Saturated Fats <10% (*n* = 72)	Saturated Fats ≥10% (*n* = 21)	Saturated Fats <7% (*n* = 34)	Saturated Fats ≥7% (*n* = 59)
HOMA-IR < 3.42	38	7	21	24
HOMA-IR ≥ 3.42	34	14	13	35

**Table 9:** Multivariable model predicting HOMA-β, F (1, 91) = 6.823, *p* = 0.011, adj. R^2^ = 0.060. *p*-values ≤ 0.050 are shown in bold.

	B	Std. Error	t	Sig.
(Constant)	1.894	0.234	8.083	**<0.001**
Total fats, g (log)	0.321	0.123	2.612	**0.011**

**Table 10:** Multivariable model predicting the QUICK index, F (2, 90) = 7.271, *p* = 0.001, adj. R^2^ = 0.120. *p*-values ≤ 0.050 are shown in bold.

	B	Std. Error	t	Sig.
(Constant)	−0.234	0.081	−2.906	**0.005**
Age, years (log)	−0.196	0.073	−2.664	**0.009**
Saturated fats, g (log)	−0.042	0.017	−2.547	**0.013**

## References

[1] Arroyo-Johnson C., Mincey K.D. (2016). Obesity Epidemiology Worldwide. Gastroenterol. Clin. N. Am..

[2] Tremmel M., Gerdtham U.-G., Nilsson P., Saha S. (2017). Economic Burden of Obesity: A Systematic Literature Review. Int. J. Environ. Res. Public Health.

[3] Finkelstein E.A., Graham W.C.K., Malhotra R. (2014). Lifetime direct medical costs of childhood obesity. Pediatrics.

[4] Ng M., Fleming T., Robinson M., Thomson B., Graetz N., Margono C., Mullany E.C., Biryukov S., Abbafati C., Abera S.F. (2014). Global, regional, and national prevalence of overweight and obesity in children and adults during 1980–2013: A systematic analysis for the Global Burden of Disease Study 2013. Lancet.

[5] Kopp W. (2019). How Western Diet And Lifestyle Drive The Pandemic Of Obesity And Civilization Diseases. Diabetes Metab. Syndr. Obes. Targets Ther..

[6] Rito A.I., Buoncristiano M., Spinelli A., Salanave B., Kunešová M., Hejgaard T., García Solano M., Fijałkowska A., Sturua L., Hyska J. (2019). Association between Characteristics at Birth, Breastfeeding and Obesity in 22 Countries: The WHO European Childhood Obesity Surveillance Initiative–COSI 2015/2017. Obes. Facts.

[7] Engin A. (2017). The Definition and Prevalence of Obesity and Metabolic Syndrome.

[8] AL M., EV K., PW Y. (2012). Prevalence of Cardiovascular Disease Risk Factors Among US Adolescents, 1999–2008. Pediatrics.

[9] Zimmet P., Alberti K.G.M., Kaufman F., Tajima N., Silink M., Arslanian S., Wong G., Bennett P., Shaw J., Caprio S. (2007). The metabolic syndrome in children and adolescents—An IDF consensus report. Pediatr. Diabetes.

[10] Mottillo S., Filion K.B., Genest J., Joseph L., Pilote L., Poirier P., Rinfret S., Schiffrin E.L., Eisenberg M.J. (2010). The metabolic syndrome and cardiovascular risk a systematic review and meta-analysis. J. Am. Coll. Cardiol..

[11] Valerio G., Maffeis C., Saggese G., Ambruzzi M.A., Balsamo A., Bellone S., Bergamini M., Bernasconi S., Bona G., Calcaterra V. (2018). Diagnosis, treatment and prevention of pediatric obesity: Consensus position statement of the Italian Society for Pediatric Endocrinology and Diabetology and the Italian Society of Pediatrics. Ital. J. Pediatr..

[12] Katzmarzyk P.T., Barlow S., Bouchard C., Catalano P.M., Hsia D.S., Inge T.H., Lovelady C., Raynor H., Redman L.M., Staiano A.E. (2014). An evolving scientific basis for the prevention and treatment of pediatric obesity. Int. J. Obes..

[13] Al-Khudairy L., Loveman E., Colquitt J.L., Mead E., Johnson R.E., Fraser H., Olajide J., Murphy M., Velho R.M., O’Malley C. (2017). Diet, physical activity and behavioural interventions for the treatment of overweight or obese adolescents aged 12 to 17 years. Cochrane Database Syst. Rev..

[14] (2014). WHO|BMI-for-Age. WHO. www.who.int/childgrowth/standards/bmi_for_age/en.

[15] World Health Organization (2011). Waist Circumference and Waist-Hip Ratio: Report of a WHO Expert Consultation.

[16] McCarthy H.D., Jarrett K.V., Crawley H.F. (2001). The development of waist circumference percentiles in British children aged 5.0–16.9 y. Eur. J. Clin. Nutr..

[17] Moreno L.A., Rodríguez G., Guillén J., Rabanaque M.J., León J.F., Ariño A. (2002). Anthropometric measurements in both sides of the body in the assessment of nutritional status in prepubertal children. Eur. J. Clin. Nutr..

[18] Yoo E.-G. (2016). Waist-to-height ratio as a screening tool for obesity and cardiometabolic risk. Korean J. Pediatr..

[19] Santoro N., Amato A., Grandone A., Brienza C., Savarese P., Tartaglione N., Marzuillo P., Perrone L., Miraglia Del Giudice E. (2013). Predicting metabolic syndrome in obese children and adolescents: Look, measure and ask. Obes. Facts.

[20] Völgyi E., Tylavsky F.A., Lyytikäinen A., Suominen H., Alén M., Cheng S. (2008). Assessing body composition with DXA and bioimpedance: Effects of obesity, physical activity, and age. Obesity.

[21] Flynn J.T., Kaelber D.C., Baker-Smith C.M., Blowey D., Carroll A.E., Daniels S.R., de Ferranti S.D., Dionne J.M., Falkner B., Flinn S.K. (2017). Clinical Practice Guideline for Screening and Management of High Blood Pressure in Children and Adolescents. Pediatrics.

[22] Expert Panel on Integrated Guidelines for Cardiovascular Health and Risk Reduction in Children and Adolescents, National Heart, Lung, and Blood Institute (2011). Expert panel on integrated guidelines for cardiovascular health and risk reduction in children and adolescents: Summary report. Pediatrics.

[23] Block G., Hartman A.M., Dresser C.M., Carroll M.D., Gannon J., Gardner L. (1986). A data-based approach to diet questionnaire design and testing. Am. J. Epidemiol..

[24] Kolodziejczyk J.K., Merchant G., Norman G.J. (2012). Reliability and validity of child/adolescent food frequency questionnaires that assess foods and/or food groups. J. Pediatr. Gastroenterol. Nutr..

[25] Società Italiana di Nutrizione Umana LARN Livelli di Assunzione di Riferimento di Nutrienti ed Energia Per La Popolazione Italiana.

[26] Shashaj B., Luciano R., Contoli B., Morino G.S., Spreghini M.R., Rustico C., Sforza R.W., Dallapiccola B., Manco M. (2016). Reference ranges of HOMA-IR in normal-weight and obese young Caucasians. Acta Diabetol..

[27] d’Annunzio G., Vanelli M., Pistorio A., Minuto N., Bergamino L., Iafusco D., Lorini R., Banin P., Cardella F., Diabetes Study Group of the Italian Society for Pediatric Endocrinology and Diabetes (2009). Insulin resistance and secretion indexes in healthy Italian children and adolescents: A multicentre study. Acta Biomed..

[28] Matthews D.R., Hosker J.P., Rudenski A.S., Naylor B.A., Treacher D.F., Turner R.C. (1985). Homeostasis Model Assessment: Insulin Resistance and Beta-Cell Function From Fasting Plasma Glucose and Insulin Concentrations in Man. Diabetologia.

[29] Brown R.J., Yanovski J.A. (2014). Estimation of insulin sensitivity in children: Methods, measures and controversies. Pediatr. Diabetes.

[30] Katz A., Nambi S.S., Mather K., Baron A.D., Follmann D.A., Sullivan G., Quon M.J. (2000). Quantitative Insulin Sensitivity Check Index: A Simple, Accurate Method for Assessing Insulin Sensitivity in Humans. J. Clin. Endocrinol. Metab..

[31] Placzkowska S., Pawlik-Sobecka L., Kokot I., Piwowar A. (2019). Indirect Insulin Resistance Detection: Current Clinical Trends and Laboratory Limitations. Biomed. Pap. Med. Fac. Palacky Univ. Olomouc.

[32] Calcaterra V., Montalbano C., de Silvestri A., Pelizzo G., Regalbuto C., Paganelli V., Albertini R., Cave F.D., Larizza D., Cena H. (2019). Triglyceride Glucose Index as a Surrogate Measure of Insulin Sensitivity in a Caucasian Pediatric Population. J. Clin. Res. Pediatr. Endocrinol..

[33] Manco M., Grugni G., Di Pietro M., Balsamo A., Di Candia S., Morino G.S., Franzese A., Di Bonito P., Maffeis C., Valerio G. (2016). Triglycerides-to-HDL cholesterol ratio as screening tool for impaired glucose tolerance in obese children and adolescents. Acta Diabetol..

[34] Di Bonito P., Moio N., Scilla C., Cavuto L., Sibilio G., Sanguigno E., Forziato C., Saitta F., Iardino R.E., Capaldo B. (2012). Usefulness of the High triglyceride-to-HDL Cholesterol Ratio to Identify Cardiometabolic Risk Factors and Preclinical Signs of Organ Damage in Outpatient Children. Diabetes Care.

[35] Niroumand S., Khajedaluee M., Khadem-Rezaiyan M., Abrishami M., Juya M., Khodaee G., Dadgarmoghaddam M. (2015). Atherogenic Index of Plasma (AIP): A marker of cardiovascular disease. Med. J. Islam. Repub. Iran.

[36] Ejtahed H.S., Kelishadi R., Hasani-Ranjbar S., Angoorani P., Motlagh M.E., Shafiee G., Ziaodini H., Taheri M., Qorbani M., Heshmat R. (2019). Discriminatory Ability of Visceral Adiposity Index as an Indicator for Modeling Cardio-Metabolic Risk Factors in Pediatric Population: The CASPIAN-V Study. J. Cardiovasc. Thorac. Res..

[37] Ashwell M., Gibson S. (2016). Waist-to-height ratio as an indicator of ‘early health risk’: Simpler and more predictive than using a ‘matrix’ based on BMI and waist circumference. BMJ Open.

[38] Barlow S.E., Dietz W.H. (1998). Obesity evaluation and treatment: Expert Committee recommendations. The Maternal and Child Health Bureau, Health Resources and Services Administration and the Department of Health and Human Services. Pediatrics.

[39] McCarthy H.D., Cole T.J., Fry T., Jebb S.A., Prentice A.M. (2006). Body fat reference curves for children. Int. J. Obes..

[40] Robinson J.G., Goldberg A.C. (2011). Treatment of Adults With Familial Hypercholesterolemia and Evidence for Treatment: Recommendations From the National Lipid Association Expert Panel on Familial Hypercholesterolemia. J. Clin. Lipidol..

[41] Zaki M.E., El-Bassyouni H.T., El-Gammal M., Kamal S. (2015). Indicators of the metabolic syndrome in obese adolescents. Arch. Med. Sci..

[42] Jaber L.A., Brown M.B., Hammad A., Zhu Q., Herman W.H. (2004). The prevalence of the metabolic syndrome among arab americans. Diabetes Care.

[43] Bustos P., Saez K., Gleisner A., Ulloa N., Calvo C., Asenjo S. (2010). Metabolic syndrome in obese adolescents. Pediatr. Diabetes.

[44] Bussler S., Penke M., Flemming G., Elhassan Y.S., Kratzsch J., Sergeyev E., Lipek T., Vogel M., Spielau U., Körner A. (2017). Novel Insights in the Metabolic Syndrome in Childhood and Adolescence. Horm. Res. Paediatr..

[45] Gidding S.S., Dennison B.A., Birch L.L., Daniels S.R., Gillman M.W., Gilman M.W., Lichtenstein A.H., Rattay K.T., Steinberger J., Stettler N. (2005). Dietary recommendations for children and adolescents: A guide for practitioners: Consensus statement from the American Heart Association. Circulation.

[46] Gow M.L., Ho M., Burrows T.L., Baur L.A., Stewart L., Hutchesson M.J., Cowell C.T., Collins C.E., Garnett S.P. (2014). Impact of Dietary Macronutrient Distribution on BMI and Cardiometabolic Outcomes in Overweight and Obese Children and Adolescents: A Systematic Review. Nutr. Rev..

[47] Sartorius K., Sartorius B., Madiba T.E., Stefan C. (2018). Does high-carbohydrate intake lead to increased risk of obesity? A systematic review and meta-analysis. BMJ Open.

[48] Jakobsen M.U., Dethlefsen C., Joensen A.M., Stegger J., Tjønneland A., Schmidt E.B., Overvad K. (2010). Intake of carbohydrates compared with intake of saturated fatty acids and risk of myocardial infarction: Importance of the glycemic index. Am. J. Clin. Nutr..

[49] del Mar Bibiloni M., Maffeis C., Llompart I., Pons A., Tur J.A. (2013). Dietary factors associated with subclinical inflammation among girls. Eur. J. Clin. Nutr..

[50] Shivappa N., Hebert J.R., Marcos A., Diaz L.-E., Gomez S., Nova E., Michels N., Arouca A., González-Gil E., Frederic G. (2017). Association between dietary inflammatory index and inflammatory markers in the HELENA study. Mol. Nutr. Food Res..

[51] Appannah G., Pot G.K., Huang R.C., Oddy W.H., Beilin L.J., Mori T.A., Jebb S.A., Ambrosini G.L. (2015). Identification of a Dietary Pattern Associated With Greater Cardiometabolic Risk in Adolescence. Nutr. Metab. Cardiovasc. Dis..

[52] Andrade M.I.S.D., Oliveira J.S., Leal V.S., Lima N.M.D.S., Bezerra P.B., Santiago E.R.C., Lira P.I.C.D. (2020). Prevalence of insulin resistance and association with metabolic risk factors and food consumption in adolescents-recife/Brazil. Rev. Paul. Pediatr..

[53] Knowler W.C., Barrett-Connor E., Fowler S.E., Hamman R.F., Lachin J.M., Walker E.A., Nathan D.M., Diabetes Prevention Program Research Group (2002). Reduction in the incidence of type 2 diabetes with lifestyle intervention or metformin. N. Engl. J. Med..

[54] Tuomilehto J., Lindström J., Eriksson J.G., Valle T.T., Hämäläinen H., Ilanne-Parikka P., Keinänen-Kiukaanniemi S., Laakso M., Salminen V., Louheranta A. (2001). Prevention of Type 2 Diabetes Mellitus by Changes in Lifestyle Among Subjects With Impaired Glucose Tolerance. N. Engl. J. Med..

[55] Lindström J., Ilanne-Parikka P., Peltonen M., Aunola S., Eriksson J.G., Hemiö K., Hämäläinen H., Härkönen P., Keinänen-Kiukaanniemi S., Laakso M. (2006). Sustained reduction in the incidence of type 2 diabetes by lifestyle intervention: Follow-up of the Finnish Diabetes Prevention Study. Lancet.

[56] Lindström J., Louheranta A., Mannelin M., Rastas M., Salminen V., Eriksson J., Uusitupa M., Tuomilehto J., Finnish Diabetes Prevention Study Group (2003). The Finnish Diabetes Prevention Study (DPS): Lifestyle intervention and 3-year results on diet and physical activity. Diabetes Care.

[57] de Souza R.J., Mente A., Maroleanu A., Cozma A.I., Ha V., Kishibe T., Uleryk E., Budylowski P., Schünemann H., Beyene J. (2015). Intake of saturated and trans unsaturated fatty acids and risk of all cause mortality, cardiovascular disease, and type 2 diabetes: Systematic review and meta-analysis of observational studies. BMJ.

